# Potential causal effect of physical activity on reducing the risk of dementia: a 6-year cohort study from the Japan Gerontological Evaluation Study

**DOI:** 10.1186/s12966-021-01212-w

**Published:** 2021-10-29

**Authors:** Koryu Sato, Naoki Kondo, Masamichi Hanazato, Taishi Tsuji, Katsunori Kondo

**Affiliations:** 1 Department of Social Epidemiology, Graduate School of Medicine and School of Public Health, Kyoto University, Floor 2, Science Frontier Laboratory, Yoshida-Konoecho, Sakyo-ku, Kyoto-shi, Kyoto, 606-8315 Japan; 2 Department of Environmental Preventive Medical Sciences, Center for Preventive Medical Sciences, Chiba University, 1-33 Yayoi-cho, Inage-ku, Chiba 263-8522 Chiba-shi, Japan; 3 Faculty of Health and Sport Sciences, University of Tsukuba, 3-29-1 Otsuka, Bunkyo-ku, Tokyo 112-0012 Japan; 4 Department of Social Preventive Medical Sciences, Center for Preventive Medical Sciences, Chiba University, 1-33 Yayoi-cho, Inage-ku, Chiba-shi, Chiba 263-8522 Japan; 5 Department of Gerontological Evaluation, Center for Gerontology and Social Science, National Center for Geriatrics and Gerontology, 7-430 Morioka-cho, Obu-shi, Aichi 474-8511 Japan

**Keywords:** Physical activity, Dementia, Instrumental variable, Snow, Japanese older adults

## Abstract

**Background:**

The causal effect of physical activity on reducing dementia risk has been questioned due to the possibility of reverse causation. This study examined the potential causal effects of physical activity on reducing dementia risk using residency in a snowy area as an instrumental variable (IV) representing the physical activity of older adults.

**Methods:**

We used cohort data from the Japan Gerontological Evaluation Study, a longitudinal cohort enrolling people aged 65 or older who were physically and cognitively independent in 2013; study participants were followed for an average of 5.7 years. Participants in the present study included 73,260 individuals living in 19 municipalities in Japan. Physical activity was measured by self-report questionnaires and the incidence of dementia was ascertained by linking participants to the public registries of long-term care insurance. IV estimation was obtained from a piecewise Cox proportional hazard model using a two-stage regression procedure.

**Results:**

During the study period, we ascertained 8714 cases (11.9%) of dementia onset. In the IV analysis, we found that the frequency of physical activity per week was negatively associated with dementia risk, though the association weakened over time (Year 1: hazard ratio = 0.53, 95% confidence interval: 0.39–0.74; Year 4: 0.69, 0.53–0.90; Year 6: 0.85, 0.66–1.10).

**Conclusions:**

Our IV analysis indicated a potential causal effect of physical activity on reducing dementia risk that persisted for at least 4 years of follow-up. Thus, we conclude that physical activity should be recommended for older adults to reduce dementia risk.

**Supplementary Information:**

The online version contains supplementary material available at 10.1186/s12966-021-01212-w.

## Introduction

As of 2015, the World Health Organization estimated that approximately 50 million people had dementia worldwide; that number is expected to triple by 2050 as the population ages [1]. Given that there is no cure for dementia, it has recommended regular physical activity to delay its onset [2]. However, despite evidence from observational epidemiologic studies, there is no clear evidence that supports a direct causal effect of physical activity on reducing dementia risk [3, 4].

A meta-analysis of randomized controlled trials (RCTs) found no evidence that physical activity interventions reduced dementia risk [4]. However, RCTs tend to have short intervention periods and small sample sizes; thus, they often lack the statistical power to detect beneficial intervention effects. In one of the RCTs which included 1635 participants who were followed-up for 24 months, no difference was found between participating in either a moderate-intensity physical activity intervention or a health education intervention and the incidence of dementia [5]. In contrast, a number of reviews and meta-analyses of prospective cohort studies have reported that physical activity is inversely associated with dementia and Alzheimer’s disease risk [6–8]. However, observational studies can be biased due to reverse causation, as levels of physical activity frequently decline in the prodromal stage of dementia [9, 10]. One possible method to address reverse causation is to follow participants for a sufficient duration and to exclude cases ascertained in the initial stage of follow-up (i.e., cases who were probably in the prodromal stage of dementia at study enrollment). A recent meta-analysis excluded individuals who experienced dementia onset within 10 years of follow-up and found no associations between physical inactivity and increased dementia risk [11]; the included studies measured physical activity only once (more than 10 years ago) and failed to consider the time-varying effects of physical activity at baseline. Another cohort study showed that the association between physical activity at baseline and dementia risk weakens over time [12]. Thus, the exclusion approach has been shown to underestimate negative associations between physical activity and dementia risk, and another approach is necessary to examine causal effects.

In this study, we used data from the Japan Gerontological Evaluation Study (JAGES) to explore the effects of physical activity on dementia risk. To address potential reverse causation and to examine the causal effects of physical activity, we applied the instrumental variable (IV) method [13]. Briefly, if there is an appropriate IV (i.e., a variable that exogenously determines treatment levels, thus assuring exchangeability between treatment and control groups and thereby simulating RCTs), this can eliminate reverse causation bias and enable causal inferences for the association between physical activity and dementia. Residency in a snowy area could be an acceptable IV for physical activity, given that snowfall hinders older adults from physical activity but does not directly affect dementia onset [14, 15]. In snowy areas, there are fewer opportunities to exercise outside, and older people who fear falling due to snow may avoid going to indoor exercise facilities. To our knowledge, this is the first study examining the causality of the association between physical activity and dementia risk using the IV method.

## Methods

### Baseline survey

**Fig. 1:**
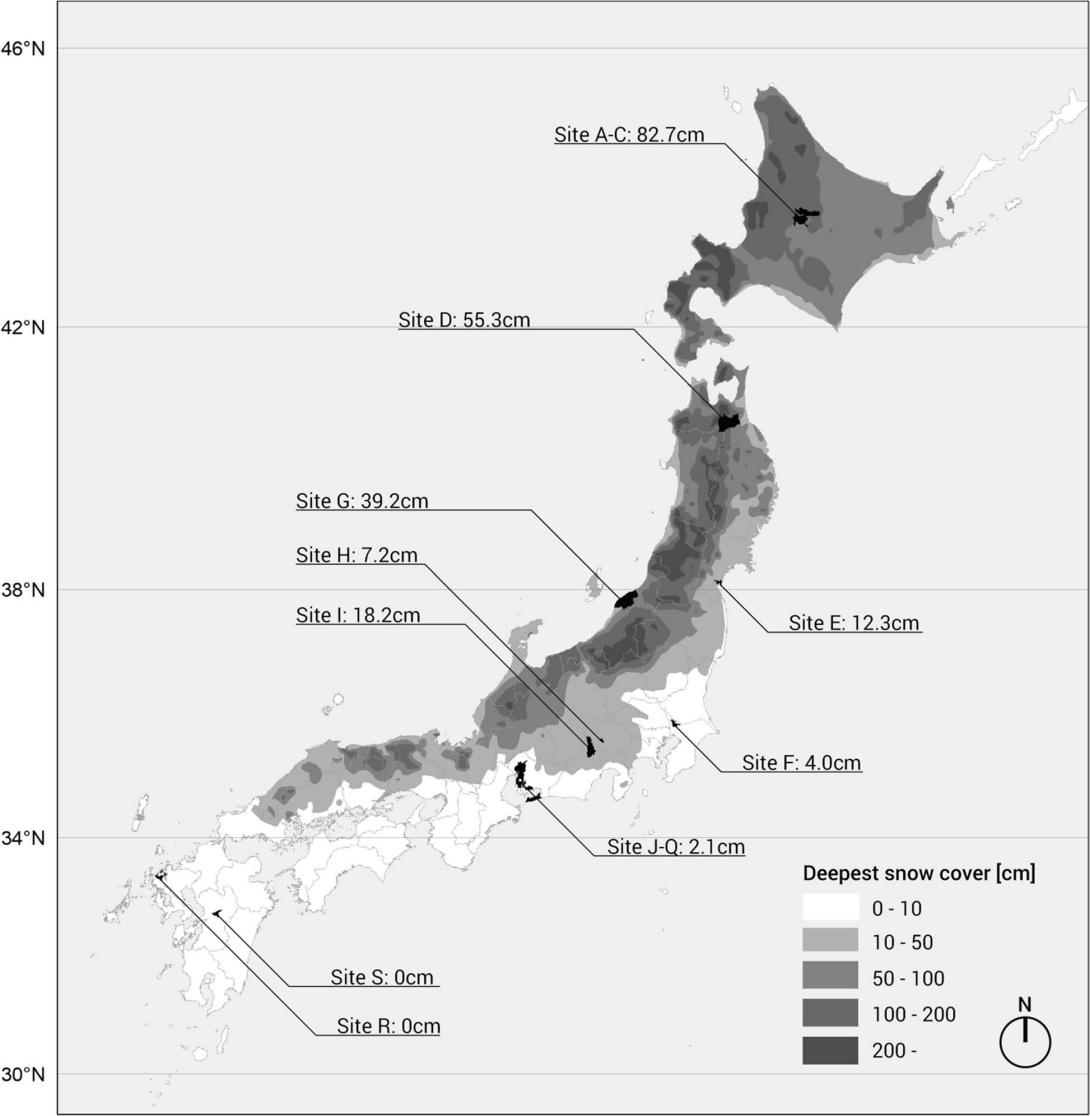
Study sites and the average of the deepest snow cover. The averages of the deepest snow cover of habitable areas were indicated for each study site, while the gradation was generated based on a deepest snow cover including inhabitable areas. Hence, the values of the average and legends do not match in some sites

The JAGES is an ongoing nationwide cohort study of Japanese people aged 65 or older who are physically and cognitively independent (i.e., not certified as needing assistance from public long-term care insurance [LTCI]; thus, those with dementia at baseline were not eligible). From October to December 2013, self-report questionnaires were mailed to eligible residents in 19 municipalities in nine (out of 47) prefectures. As regions in northern and southern Japan are far apart, we could take advantage of the variability in snowfall across the country and obtain data on older adults living in snowy and non-snowy areas (Fig. 1). Random sampling methods were used in ten large municipalities, while a census of all eligible residents was conducted in nine smaller municipalities. Of 112,705 people invited to participate, 79,291 people returned the questionnaires (response rate = 70.4%). A total of 4994 respondents whose sex and age were erroneously recorded or could not be confirmed were excluded from the current study. This study was reviewed and approved by the ethics committees at Nihon Fukushi University (13–14), Chiba University (3442), and Kyoto University (R3153) and was conducted in accordance with the principles of the Declaration of Helsinki and its later amendments.

### Dementia incidence

**Fig. 2:**
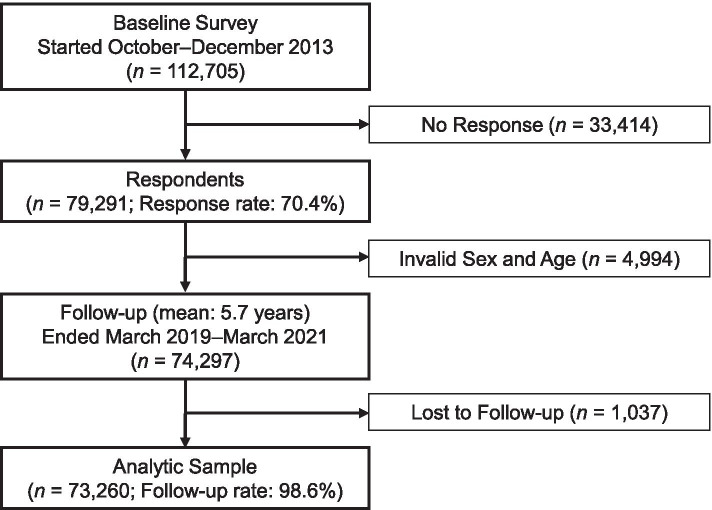
A flow-chart of the analytic sample

We ascertained dementia incidence by linking participants to the LTCI registries administrated by each municipality. The follow-up period started between October and November 2013 and ended between March 2019 and March 2021 (mean follow-up, 5.7 years). Of the eligible sample (74,297 individuals), 73,260 participants were successfully linked to administrative records (follow-up rate = 98.6%). Figure 2 shows a flow-chart of our analytic sample.

In Japan, all citizens aged 40 or older enroll in the LTCI system and receive benefits if they are certified as needing long-term care [16]. The eligibility criteria and benefits are nationally standardized based on the Long-Term Care Insurance Act. In the assessment, trained investigators visit applicants’ homes to assess their eligibility using a standardized protocol and classify applicants into one of seven levels of cognitive impairment (Supplementary Table A); this scale has been validated against gold-standard assessment tools: the Mini-Mental State Examination and the Clinical Dementia Rating [17, 18]. We defined level II (i.e. ‘manifests some symptoms/behaviors and communication difficulties that may hinder daily activities, but can be independent if someone takes care them’) or higher on the cognitive impairment scale as dementia, according to guidelines from the Japanese Ministry of Health, Labor and Welfare and previous studies [19, 20]. If applicants are certified as having dementia, the public LTCI registry records the date of certification. During the study period, we ascertained incident dementia in 8714 (11.9%) cases.

### Physical activity

The primary study exposure was the frequency of physical activity per week. We measured the frequency of vigorous, moderate, and light intensity physical activity separately with a self-report questionnaire developed based on the English Longitudinal Study of Ageing; this physical activity measure has been objectively validated and has demonstrated face validity in predicting various health outcomes [21–23]. Participants were asked, ‘How often do you exert yourself (vigorously/moderately/lightly) in the following activities?;’ vigorous exercise was exemplified by ‘running, swimming, cycling, tennis, exercise at the gym, mountain climbing,’ moderate exercise was exemplified by ‘walking (at a brisk pace), dancing, gymnastics, golf, farming, gardening, car washing,’ and light exercise was exemplified by ‘stretching (calisthenics), bowling, walking to shops or the station, laundry.’ The frequency of each activity was measured using a six-option scale and we assigned specific values for each option based on the number physical activity sessions per week as follows: 5.5 = four or more times per week, 2.5 = two or three times per week, 1 = once per week, 0.5 = one to three times per month, 0.1 = a few times per year, and 0 = rarely. To integrate the three different levels of physical activity into one indicator, we weighted the responses based on metabolic equivalents of task (METs). We referred to the International Physical Activity Questionnaire and the Global Physical Activity Questionnaire, which assign 8.0 METs for vigorous physical activity and 4.0 METs for moderate physical activity, but do not define METs for light physical activity [24, 25]; we assigned 1.5 METs for light physical activity because sedentary behavior is often defined as physical activity below 1.5 METs [26] and because the Japan Public Health Center Based Prospective Study had previously assigned 1.5 METs for ‘other activities’ (a category equivalent to light physical activity) [27]. Hence, the physical activity index was calculated as follows:

$$Physical\ Activity\ (PA)\ Index=\frac{\left(8.0\ast the\ frequency\ of\ vigorous\ PA+4.0\ast moderate\ PA+1.5\ast light\ PA\right)}{13.5}$$

### Instrumental variable

We treated residency in a snowy area as an IV for physical activity, with the assumption that residency in a snowy area would be negatively correlated with the frequency of physical activity. We consulted the National Land Numerical Information complied by the Ministry of Land, Infrastructure, Transport and Tourism in 2012 to obtain data on the deepest snow cover (i.e., a 30-year average of the annual highest snowfall in a 1 km mesh unit) in habitable areas of 467 school districts within the 19 municipalities. We defined a school district with an average deepest snow cover of 10 cm (3.94 in) or more as a snowy area. The snow cover exceeds 10 cm for about 4 months in sites A-C in Fig. 1, about 2 months in site B, and for less than 1 month in other defined snowy areas.

### Covariates

We adjusted for potential confounders measured at baseline: sex, age, educational attainment (low: ≤9 years, middle: 10–12 years, high: ≥13 years), annual equivalized household income (low: < 2.0 million Japanese Yen [MJPY], middle: 2.0–4.0 MJPY, high: ≥4.0 MJPY) [28], the existence of cardiometabolic disease (heart disease, stroke, diabetes) [11], depressive symptoms assessed with the 15-item Geriatric Depression Scale (not depressed: ≤4 points, moderately depressed: 5–9 points, severely depressed: ≥10 points) [29], drinking habits (drinker or not), and smoking status (smoker or not). In addition, we adjusted for the following binary (yes/no) variables representing social relationships based on results from a previous study: married, engaged in paid work, contact with friends once a month or more, participating in community groups (e.g., volunteering, sports groups, hobby groups, other community groups), and exchanging family support (i.e., spouse or children living together) as defined by a positive answer to at least one of the following four questions: (i) ‘Do your family members listen to your concerns and complaints?,’ (ii) ‘Do you listen to your family members’ concerns and complaints?,’ (iii) ‘Do your family members look after you when you are sick and confined to a bed for a few days?,’ and (iv) ‘Do you look after your family members when they are sick and confined to a bed for a few days?’) [20]. We also controlled for potential confounders at the school-district level: population density, average degree of slopes, and annual hours of sunlight.

### Statistical analysis

We applied the IV method to a Cox proportional hazard model using a two-stage regression approach (see the Supplemental Appendix of Tchetgen Tchetgen et al. [30] for the proof). We assumed that the underlying failure time outcome follows a Cox proportional hazard model:

1 $$\boldsymbol{h}\left(\boldsymbol{t}|\boldsymbol{A},\boldsymbol{U},\boldsymbol{Z}\right)={\boldsymbol{h}}_{\mathbf{0}}\left(\boldsymbol{t}\right)\mathbf{\exp}\left({\boldsymbol{b}}_{\boldsymbol{a}}\boldsymbol{A}+{\boldsymbol{b}}_{\boldsymbol{u}}\left(\boldsymbol{U},\boldsymbol{t}\right)\right)$$

where *h*(*t*| ∙) is a conditional hazard function at time *t*, *A* is a treatment (i.e., physical activity), U is an unobserved confounder (i.e., a potential source of reverse causality, such as the prodromal stage of dementia), Z is an IV (i.e. residency in a snowy area), *h*_0_(*t*) is a baseline hazard function, b_a_ is the log-hazards ratio encoding the effect of the treatment, and b_u_ is the effect of the unobserved confounder at time t. We cannot directly estimate the equation above due to the existence of the unobserved variable *U*, as b_a_ can be biased if we ignore *U*. However, if there is an appropriate IV, we can obtain an unbiased estimate of b_a_ without controlling for *U*. The IV method requires compliance with three assumptions: (i) the relevance condition (Z is associated with A), (ii) the exclusion restriction (Z affects the outcome [i.e., dementia] only through A), and (iii) marginal exchangeability (Z and the outcome do not share causes) [13]. Here, we define the relationship between A and Z as follows:

2 $${\displaystyle \begin{array}{c}\boldsymbol{A}={\boldsymbol{c}}_{\mathbf{0}}+{\boldsymbol{c}}_{\boldsymbol{z}}\boldsymbol{Z}+\Delta \end{array}}$$

where ∆ is mean zero residual error independent of Z. With the IV assumptions and the rare disease assumption (i.e., a conditional survival curve is *S*(*t*| *A*, *U*, *Z*) ≈ 1), we can obtain a hazard function conditional on Z as follows:

3 $$\boldsymbol{h}\left(\boldsymbol{t}|\boldsymbol{Z}\right)\approx \boldsymbol{E}\left[\boldsymbol{h}\left(\boldsymbol{t}|\boldsymbol{A},\boldsymbol{U},\boldsymbol{Z}\right)|\boldsymbol{Z}\right]=\boldsymbol{E}\left[{\boldsymbol{h}}_{\mathbf{0}}\left(\boldsymbol{t}\right)\mathbf{\exp}\left({\boldsymbol{b}}_{\boldsymbol{a}}\boldsymbol{A}+{\boldsymbol{b}}_{\boldsymbol{u}}\left(\boldsymbol{U},\boldsymbol{t}\right)\right)|\boldsymbol{Z}\right]{\displaystyle \begin{array}{c}=\boldsymbol{E}\left[{\boldsymbol{h}}_{\mathbf{0}}\left(\boldsymbol{t}\right)\mathbf{\exp}\left({\boldsymbol{b}}_{\boldsymbol{a}}\left({\boldsymbol{c}}_{\mathbf{0}}+{\boldsymbol{c}}_{\boldsymbol{z}}\boldsymbol{Z}+\Delta \right)+{\boldsymbol{b}}_{\boldsymbol{u}}\left(\boldsymbol{U},\boldsymbol{t}\right)\right)|\boldsymbol{Z}\right]={\boldsymbol{h}}_{\mathbf{0}}^{\ast}\left(\boldsymbol{t}\right)\mathbf{\exp}\left({\boldsymbol{b}}_{\boldsymbol{a}}\left({\boldsymbol{c}}_{\mathbf{0}}+{\boldsymbol{c}}_{\boldsymbol{z}}\boldsymbol{Z}\right)\right)\end{array}}$$

where ${h}_0^{\ast}(t)=E\left[{\exp}\left({b}_a\Delta+{b}_u\left(U,t\right)\right)\right]{h}_0(t)$ given *U* and ∆ are independent of Z. In the two-stage regression procedure, we predict $\hat{A}$ on Z using ordinary least-squares regression as the first-stage estimation

4 $${\displaystyle \begin{array}{c}\hat{\boldsymbol{A}}=\hat{{\boldsymbol{c}}_{\mathbf{0}}}+\hat{{\boldsymbol{c}}_{\boldsymbol{z}}}\boldsymbol{Z}\end{array}}$$

Given *E*[*A*| *Z*] = *c*_0_ + *c*_*z*_*Z*, we substitute the predicted mean value of A into the Eq. (3) as the second-stage estimation

5 $${\displaystyle \begin{array}{c}\boldsymbol{h}\left(\boldsymbol{t}|\boldsymbol{Z}\right)\approx {\boldsymbol{h}}_{\mathbf{0}}^{\ast}\left(\boldsymbol{t}\right)\mathbf{\exp}\left({\boldsymbol{b}}_{\boldsymbol{a}}\hat{\boldsymbol{A}}\right)\end{array}}$$

Equation (5) expresses that we can obtain an unbiased estimate of b_a_ regardless of U using Z if the assumptions hold. In other words, we can eliminate possible reverse causation bias of the estimated effect of physical activity on dementia due to unobserved confounders (i.e., the prodromal stage of dementia) using residency in a snowy area as an IV. We predicted physical activity using residency in a snowy area and the above covariates in the first-stage estimation. In the second-stage estimation, we included predicted physical activity, covariates, and fixed effects of municipalities to adjust for unobserved regional characteristics.

An ordinary Cox proportional hazard model assumes that hazard ratios are constant through the study period. Nevertheless, we assumed that the effect of physical activity at baseline would be attenuated over time. Indeed, we found that the variable of physical activity was interacting with time, indicating that the proportional hazards assumption was not satisfied. Therefore, we split the study period into one-year periods and estimated hazard ratios for each year. Missing values were imputed using multiple imputation by chained equations, which provides similar predictions to multivariate normal imputation [31], to handle discrete variables. We generated ten imputed datasets and merged them, assuming that the data were missing at random (i.e., a missing mechanism is related to other variables measured in the same survey for that individual). Standard errors for an IV estimation were obtained by bootstrapping with 100 replications for each imputed dataset. All analyses were performed using STATA software, version 16.1 (Stata Corp, College Station, TX, US).

## Results

Participant characteristics are presented in Table 1. Of the participants enrolled in this study, 55,188 (75.3%) lived in a non-snowy area and 18,072 lived in a snowy area. We compared the characteristics of residents between non-snowy and snowy areas using standardized differences (in general, a value < 0.1 indicates balance between the two groups [32]). We confirmed that the participant characteristics were well-balanced, with the exception of mean age, household income, and frequency of physical activity; residents in a non-snowy area were younger (73.7 vs. 74.4 years), had a higher household income (8.8% vs. 6.3% in the highest category), and engaged in physical activity more frequently (1.38 vs. 1.25 times per week). We ascertained 6218 (11.3%) and 2496 (13.8%) cases of dementia onset in non-snowy areas and snowy areas, respectively.

**Table 1:** Characteristics of the participants (*N* = 73,260)

	**Non-snowy area (*n* = 55,188)**		**Snowy area (*n* = 18,072)**		**Standardized differences**^**a**^
	**n**	**%**	**n**	**%**	
Men	25,924	47.0	8107	44.9	−0.05
Age, years (mean [SD])	73.7	0.03	74.4	0.05	0.13
Education					−0.09
Low	22,327	40.5	8175	45.2	
Middle	20,549	37.2	6411	35.5	
High	11,121	20.2	3044	16.8	
Missing or other	1191	2.2	442	2.4	
Household income					−0.17
Low	22,572	40.9	8413	46.6	
Middle	17,332	31.4	4905	27.1	
High	4851	8.8	1132	6.3	
Missing	10,433	18.9	3622	20.0	
Married	39,257	71.1	12,405	68.6	−0.05
Missing	1325	2.4	574	3.2	
Engaging in paid work	11,939	21.6	3646	20.2	−0.04
Missing	5324	9.6	1738	9.6	
Cardiometabolic diseases					
Heart disease	5653	10.2	2106	11.7	0.03
Stroke	1657	3.0	647	3.6	0.04
Diabetes	7069	12.8	2354	13.0	0.02
Missing	3768	6.8	1023	5.7	
Depressive symptoms					0.09
Not depressed	34,218	62.0	10,539	58.3	
Moderately depressed	8564	15.5	3309	18.3	
Severely depressed	2849	5.2	1052	5.8	
Missing	9557	17.3	3172	17.6	
Drinker	19,049	34.5	6531	36.1	0.02
Missing	910	1.6	263	1.5	
Smoker	5536	10.0	1787	9.9	0.00
Missing	979	1.8	320	1.8	
Exchanging support with family	45,128	81.8	14,627	80.9	−0.02
Missing	656	1.2	205	1.1	
Having contact with friends	38,167	69.2	12,478	69.0	− 0.02
Missing	2976	5.4	895	5.0	
Participating in community groups	37,686	68.3	12,834	71.0	0.03
Missing	5462	9.9	1487	8.2	
The frequency of physical activity per week, times (mean [SD])	1.38	0.01	1.25	0.01	−0.11
Missing	9806	17.8	3159	17.5	
Dementia incidence	6218	11.3	2496	13.8	0.09

^a^In general, standardized differences less than 0.1 indicate balance between the two groups

Table 2 shows results from piecewise Cox proportional hazard models for the association between physical activity and dementia onset. In the conventional analysis (i.e., a standard multivariable model), the frequency of physical activity per week was negatively associated with dementia risk (Year 1: hazard ratio = 0.75, 95% confidence interval: 0.69–0.82; Year 2: 0.79, 0.74–0.84; Year 3: 0.86, 0.81–0.91; Year 4: 0.90, 0.85–0.95; Year 5: 0.94, 0.90–0.99; Year 6: 0.95, 0.92–0.99). For the IV analysis, we confirmed that residency in a snowy area was negatively associated with physical activity (coefficient = − 0.19, 95% CI: − 0.23–-0.15, *P*-value < 0.001); its partial F-statistic was 80.1 at the first-stage estimation, indicating a strong association (Supplementary Table B). In Table 2, our IV analysis showed negative associations clearly through Year 4, though the association became vague at Year 6 (Year 1: 0.53, 0.39–0.74; Year 2: 0.63, 0.49–0.82; Year 3: 0.75, 0.58–0.96; Year 4: 0.69, 0.53–0.90; Year 5: 0.82, 0.63–1.05; Year 6: 0.85, 0.66–1.10).

**Table 2:** Cox regressions for the association between physical activity and dementia

	Conventional analysis			Instrumental variable analysis		
	HR	95% CI		HR	95% CI	
Year 1	0.75	0.69	0.82	0.53	0.39	0.74
Year 2	0.79	0.74	0.84	0.63	0.49	0.82
Year 3	0.86	0.81	0.91	0.75	0.58	0.96
Year 4	0.90	0.85	0.95	0.69	0.53	0.90
Year 5	0.94	0.90	0.99	0.82	0.63	1.05
Year 6	0.95	0.92	0.99	0.85	0.66	1.10
F-statistic^a^	–			80.1		

All models included following covariates: sex, age, educational attainment, annual equivalized household income, marital status, paid work, the existence of heart disease, stroke, and diabetes, depressive symptoms, drinking habits, smoking status, family support, contact with friends, participation in community groups, population density, average degree of slopes, annual hours of sunlight, and fixed effects of municipalities

*Abbreviations*: *HR* Hazard ratio, *CI* Confidence interval

^a^Partial F-statistic for a snowy area at the first-stage estimation

As a sensitivity analysis, we reviewed various cut-off points for deepest snow cover (Supplementary Table C). We obtained identical results when examining other cut-offs and found that the cut-off of 10 cm (3.94 in) was most strongly associated with physical activity. To examine the robustness of estimates, we excluded participants who were censored within 1 year (71,522 participants and 7882 cases of dementia were left) and obtained similar results (Supplementary Table D). In addition, we conducted a complete case analysis (i.e., using data only from participants without missing values, *n* = 37,873) and found similar point estimates but much wider confidence intervals in the IV analysis than in the full sample analysis, probably due to the limited sample size (Supplementary Table E).

## Discussion

The present study explored the causal effect of physical activity on reducing dementia risk using an IV analysis and found that the beneficial effects of physical activity persisted for at least 4 years. Our results are consistent with the biomedical rationale for the beneficial effects of physical activity on brain health, such as improving cerebral blood flow, neurogenesis and synaptogenesis, preserving brain volume, and attenuating β-amyloid burden and tau phosphorylation [8]. However, the point estimates of the IV got close to the null over time in line with a recent large cohort study [12]. Although our IV estimates had wide confidence intervals and were not conclusive, we interpret the attenuated hazard ratios as the time-varying effects of physical activity. Some participants may continue exercising whereas others may cease during the study period. In such a situation, the physically active group at baseline could be contaminated with the inactive group over time, and the effect of physical activity measured at baseline could fade. A recent article showed that the protective effect of social participation against depressive symptoms could fade quickly once older adults stopped participation and that sustained social participation had a greater magnitude on the reduced risk of depressive symptoms than one-time participation at baseline [33]. As in this case, if the participants keep exercising, the effect of physical activity on the delayed onset of dementia may persist [6–8].

Our innovative idea of using an indicator for residency in a snowy area as an IV for physical activity is another novel contribution of this study to the field of exercise epidemiology. Many previous epidemiologic studies have implemented IVs, including distance to facilities and facility or physician treatment patterns [34]. In this work, we added a new option to use regional climate (such as snowfall) as an IV. In the social science literature, weather is often used as an IV. For example, many studies use rainfall as an IV to predict various endogenous variables, such as election turnout, electricity shortages, and income [35–37]. Rainfall fluctuates frequently, however, and thus its effects may not be consistent in the long term. On the other hand, accumulated snow continuously hinders older adults from physical activity during the snowy seasons [14, 15]. Moreover, we assumed that the effect of snowfall persists even in non-snowy seasons, as being homebound during snowy seasons can reduce muscle strength in older adults and make it difficult for them to engage in physical activity [38].

As noted above, the IV method needs to satisfy three assumptions: (i) the relevance condition, (ii) the exclusion restriction, and (iii) marginal exchangeability [13]. For assumption (i), we confirmed that residency in a snowy area was negatively associated with physical activity and that its partial F-statistic at the first-stage estimation exceeded a value of 10, indicating that the IV was strong [39]. On the other hand, assumptions (ii) and (iii) cannot be empirically verified. Assumption (ii) can be violated if the IV affects the outcome through an alternative pathway (i.e., other than through the treatment pathway). However, it is difficult to think of a plausible mechanism by which living in a snowy area would directly increase the risk of dementia. In addition, we stratified participant characteristics by the IV as recommended in previous literature [34] and confirmed that the characteristics were mostly balanced between non-snowy and snowy areas (other than the frequency of physical activity). The balance of observed characteristics across the IV allowed us to assume that unobserved characteristics were similarly balanced. Assumption (iii) can be violated if the IV shares causes with the outcome. A previous review called such a third variable ‘an instrument-outcome confounder’ and listed geographical characteristics (e.g. rurality) as potential confounders [34]. To address potential confounders, we adjusted for population density, the average degree of slopes, and annual hours of sunlight for each school district and additionally adjusted for fixed effects of municipalities in the second-stage estimation to block pathways from unobserved geographical characteristics.

The present study had several limitations. First, we may not have fully accounted for the seasonal variations of the IV. The baseline study was conducted in October in some snowy municipalities; thus, participants may have reported themselves as being more active than they were during the snowy season. Another possibility is that in heavy snowfall areas, the level of physical activity might increase due to seasonal activities such as snow shoveling and skiing. If an IV poorly predicts a treatment, the IV estimates will tend to be overstated. However, the partial F-statistic above 80 indicated that our instrument was strongly correlated with physical activity. Second, we ensured the plausibility of assumptions (ii) the exclusion restriction and (iii) the marginal exchangeability by comparing the characteristics of residents between non-snowy and snowy areas and adjusting for potential confounders in the model. However, if there were unconsidered factors that could violate the assumptions, our IV estimates would be biased. Third, the generalizability of our findings is limited. The estimand of the IV method is the local average treatment effect, and is defined as the average treatment effect for the subgroup of ‘compliers’ for the IV (those who would be less active if they lived in a snowy area) [13]. In addition, our study participants were limited to the Japanese population, though the study sites were located nationwide. Further studies conducted in other snowy countries are needed to confirm the validity of our IV and results. Fourth, some residents did not return the questionnaires, and we could not obtain information on non-respondents to address possible selection bias. However, the robust response rate of 70.4% in the baseline survey was comparable to or higher than response rates in similar studies involving community-dwelling older adults [40], and the follow-up rate of 98.6% was very high, thanks to the cooperation of the municipalities. Finally, the frequency of physical activity was measured with a self-report questionnaire, and we did not assess time spent performing physical activity. Although the physical activity measure used in this study has been objectively validated and widely used in studies conducted within the English Longitudinal Study of Ageing [21–23], reporting bias could occur; therefore, further studies with an objective measurement of physical activity are needed.

## Conclusion

In contrast to some studies reporting equivocal evidence or evidence against a causal effect of physical activity on reducing dementia risk, the present study found a potential causal effect of physical activity on reducing dementia risk that persisted for at least 4 years of follow-up. Thus, based on our study and the current literature, we conclude that physical activity should be recommended for older adults to reduce the risk of dementia.

## Supplementary Information

**Additional file 1: Table A.** Criteria for Levels of Cognitive Impairment in the Japanese Long-term Care Insurance System. **Table B.** The first-stage estimation between physical activity and residency in a snowy area. **Table C.** Comparison across different cut-offs of a deepest snow cover for the definition of a snowy area. **Table D.** Cox regressions for the association between physical activity and dementia excluding dementia onset within a year (*N* = 71,522). **Table E.** Complete case analysis using Cox regressions for the association between physical activity and dementia (*N* = 37,873).

## Data Availability

All JAGES datasets have ethical or legal restrictions for public deposition due to the inclusion of sensitive information from human participants. All enquiries are to be addressed to the data management committee via email: dataadmin.ml@jages.net.

## Abbreviations

RCT: Randomized controlled trial
IV: Instrumental variable
LTCI: Long-term care insurance
METs: Metabolic equivalents of task
MJPY: Million Japanese Yen
