# Supplementary material for: Potential causal effect of physical activity on reducing the risk of dementia: a 6-year cohort study from the Japan Gerontological Evaluation Study
Source: Int J Behav Nutr Phys Act. 2021 Oct 29;18:140. doi: 10.1186/s12966-021-01212-w (PMC8555243; doi:10.1186/s12966-021-01212-w)
Supplement: Supplementary file 1 — Additional file 1: Table A. Criteria for Levels of Cognitive Impairment in the Japanese Long-term Care Insurance System. Table B. The first-stage estimation between physical activity and residency in a snowy area. Table C. Comparison across different cut-offs of a deepest snow cover for the definition of a snowy area. Table D. Cox regressions for the association between physical activity and dementia excluding dementia onset within a year (N = 71,522). Table E. Complete case analysis using Cox regressions for the association between physical activity and dementia (N = 37,873). [file 12966_2021_1212_MOESM1_ESM.docx]

**Electronic Supplementary Material**

**Potential causal effect of physical activity on reducing the risk of dementia: A 6-year cohort study from the Japan Gerontological Evaluation Study**

**Table A. Criteria for Levels of Cognitive Impairment in the Japanese Long-term Care Insurance System**

| Rank | | Criteria | Examples of observable symptoms or behaviors |
| --- | --- | --- | --- |
| Independent | |  |  |
| I | | Suffers from a certain cognitive decline, but the daily living is almost all independent in the domestic and social spheres. |  |
| II | | Manifests some symptoms/behaviors and communication difficulties that may hinder the daily activities, but can be independent if someone takes care them. |  |
|  | IIa | The abovementioned conditions in II are observed while outside the domestic sphere. | Frequently gets lost on the street, or makes noticeable mistakes in matters that the person was previously able to handle, such as shopping, personal administrative tasks, or financial management. |
|  | IIb | The abovementioned conditions in II are also observed in the domestic sphere. | Is unable to manage taking medication or stay alone at home due to an inability to answer the phone or the door. |
| III | | Occasionally manifests communication difficulties or symptoms/behaviors that hinder daily activities, thus requiring care. |  |
|  | IIIa | Manifests abovementioned conditions described in III predominantly during the day. | Has difficulty or takes time to change clothes, take meals, defecate, or urinate; puts objects into the mouth, picks up and collects objects, is incontinent, makes loud and incoherent screams, carelessly handles fire, or engages in unhygienic acts or inappropriate sexual acts, etc. |
|  | IIIb | Manifests abovementioned conditions described in III predominantly at night. | Same as rank IIIa. |
| IV | | Frequently manifests difficulties communicating or symptoms/behaviors that hinder daily activities and constantly requires care. | Same as rank III. |

**Table B. The first-stage estimation between physical activity and residency in a snowy area**

|  | Coef. | 95% CI | | P-value |
| --- | --- | --- | --- | --- |
| Snowy area | -0.19 | -0.23 | -0.15 | <0.001 |
| Men | 0.07 | 0.05 | 0.10 | <0.001 |
| Age | -0.02 | -0.02 | -0.02 | <0.001 |
| Education |  |  |  |  |
| Middle | -0.03 | -0.05 | -0.001 | 0.04 |
| High | 0.01 | -0.02 | 0.04 | 0.56 |
| Household income |  |  |  |  |
| Middle | -0.02 | -0.04 | -0.002 | 0.03 |
| High | -0.04 | -0.07 | -0.003 | 0.04 |
| Married | 0.05 | 0.02 | 0.08 | <0.001 |
| Engaging in paid work | -0.24 | -0.27 | -0.22 | <0.001 |
| Cardiometabolic disease |  |  |  |  |
| Heart disease | -0.18 | -0.21 | -0.15 | <0.001 |
| Stroke | -0.13 | -0.19 | -0.08 | <0.001 |
| Diabetes | 0.02 | -0.01 | 0.06 | 0.13 |
| Depressive symptoms |  |  |  |  |
| Moderately depressed | -0.31 | -0.33 | -0.28 | <0.001 |
| Severely depressed | -0.45 | -0.49 | -0.41 | <0.001 |
| Drinker | 0.09 | 0.07 | 0.11 | <0.001 |
| Smoker | -0.31 | -0.34 | -0.28 | <0.001 |
| Exchanging support with family | -0.01 | -0.04 | 0.02 | 0.45 |
| Having contact with friends | 0.28 | 0.26 | 0.31 | <0.001 |
| Participating in community groups | 0.43 | 0.41 | 0.45 | <0.001 |
| Population density | -0.01 | -0.01 | -0.002 | 0.001 |
| Average degree of slopes | 0.01 | -0.003 | 0.03 | 0.11 |
| Annual hours of sunlight | -0.06 | -0.10 | -0.03 | 0.001 |
| Constant | 2.65 | 2.40 | 2.90 | <0.001 |
| Partial F-statistic for a snowy area | 80.1 | | | |

**Table C. Comparison across different cut-offs of a deepest snow cover for the definition of a snowy area**

|  | 6 cm (2.36 in) | | | 8 cm (3.15 in) | | | 10 cm (3.94 in) | | | 12 cm (4.72 in) | | | 14 cm (5.51 in) | | |
| --- | --- | --- | --- | --- | --- | --- | --- | --- | --- | --- | --- | --- | --- | --- | --- |
|  | HR | 95% CI | | HR | 95% CI | | HR | 95% CI | | HR | 95% CI | | HR | 95% CI | |
| Year 1 | 0.53 | 0.39 | 0.73 | 0.53 | 0.39 | 0.73 | 0.53 | 0.39 | 0.74 | 0.53 | 0.39 | 0.73 | 0.52 | 0.38 | 0.72 |
| Year 2 | 0.63 | 0.49 | 0.82 | 0.63 | 0.49 | 0.82 | 0.63 | 0.49 | 0.82 | 0.63 | 0.49 | 0.82 | 0.63 | 0.49 | 0.82 |
| Year 3 | 0.75 | 0.58 | 0.96 | 0.74 | 0.58 | 0.95 | 0.75 | 0.58 | 0.96 | 0.74 | 0.58 | 0.95 | 0.74 | 0.57 | 0.95 |
| Year 4 | 0.70 | 0.54 | 0.91 | 0.69 | 0.53 | 0.89 | 0.69 | 0.53 | 0.90 | 0.69 | 0.53 | 0.89 | 0.71 | 0.54 | 0.91 |
| Year 5 | 0.81 | 0.63 | 1.05 | 0.81 | 0.63 | 1.04 | 0.82 | 0.63 | 1.05 | 0.81 | 0.63 | 1.04 | 0.82 | 0.64 | 1.06 |
| Year 6 | 0.85 | 0.66 | 1.10 | 0.84 | 0.65 | 1.09 | 0.85 | 0.66 | 1.10 | 0.84 | 0.65 | 1.09 | 0.86 | 0.66 | 1.11 |
| F-statistic† | 19.0 | | | 60.0 | | | 80.1 | | | 57.1 | | | 4.6 | | |

Abbreviations: HR, hazard ratio; CI, confidence interval.

Note: All models included following covariates: sex, age, educational attainment, annual equivalized household income, marital status, paid work, the existence of heart disease, stroke, and diabetes, depressive symptoms, drinking habits, smoking status, family support, contact with friends, participation in community groups, population density, average degree of slopes, annual hours of sunlight, and fixed effects of municipalities.

† Partial F-statistic for a snowy area at the first-stage estimation.

**Table D. Cox regressions for the association between physical activity and dementia excluding dementia onset within a year (N=71,522)**

|  | Conventional analysis | | | Instrumental variable analysis | | |
| --- | --- | --- | --- | --- | --- | --- |
|  | HR | 95% CI | | HR | 95% CI | |
| Year 1 | - |  |  | - |  |  |
| Year 2 | 0.79 | 0.73 | 0.84 | 0.65 | 0.47 | 0.90 |
| Year 3 | 0.86 | 0.81 | 0.91 | 0.77 | 0.59 | 1.000 |
| Year 4 | 0.90 | 0.85 | 0.95 | 0.71 | 0.54 | 0.94 |
| Year 5 | 0.94 | 0.90 | 0.99 | 0.84 | 0.64 | 1.10 |
| Year 6 | 0.95 | 0.91 | 0.99 | 0.87 | 0.66 | 1.15 |
| F-statistic† | - | | | 76.0 | | |

Abbreviations: HR, hazard ratio; CI, confidence interval.

Note: All models included following covariates: sex, age, educational attainment, annual equivalized household income, marital status, paid work, the existence of heart disease, stroke, and diabetes, depressive symptoms, drinking habits, smoking status, family support, contact with friends, participation in community groups, population density, average degree of slopes, annual hours of sunlight, and fixed effects of municipalities.

† Partial F-statistic for a snowy area at the first-stage estimation.

**Table E. Complete case analysis using Cox regressions for the association between physical activity and dementia (N=37,873)**

|  | Conventional analysis | | | Instrumental variable analysis | | |
| --- | --- | --- | --- | --- | --- | --- |
|  | HR | 95% CI | | HR | 95% CI | |
| Year 1 | 0.73 | 0.65 | 0.82 | 0.47 | 0.04 | 5.74 |
| Year 2 | 0.82 | 0.75 | 0.89 | 0.55 | 0.05 | 6.61 |
| Year 3 | 0.82 | 0.75 | 0.89 | 0.70 | 0.06 | 8.34 |
| Year 4 | 0.89 | 0.82 | 0.96 | 0.59 | 0.05 | 7.19 |
| Year 5 | 0.93 | 0.87 | 0.99 | 0.75 | 0.06 | 9.01 |
| Year 6 | 0.95 | 0.90 | 1.002 | 0.74 | 0.06 | 8.94 |
| F-statistic† | - | | | 51.6 | | |

Abbreviations: HR, hazard ratio; CI, confidence interval.

Note: All models included following covariates: sex, age, educational attainment, annual equivalized household income, marital status, paid work, the existence of heart disease, stroke, and diabetes, depressive symptoms, drinking habits, smoking status, family support, contact with friends, participation in community groups, population density, average degree of slopes, annual hours of sunlight, and fixed effects of municipalities.

† Partial F-statistic for a snowy area at the first-stage estimation.
